# Spontaneous formation of Au–Pt alloyed nanoparticles using pure nano-counterparts as starters: a ligand and size dependent process†
†Electronic supplementary information (ESI) available: supplementary STEM and EDS data. See DOI: 10.1039/c5nr01819f



**DOI:** 10.1039/c5nr01819f

**Published:** 2015-05-19

**Authors:** Laura Usón, Victor Sebastian, Alvaro Mayoral, Jose L. Hueso, Adela Eguizabal, Manuel Arruebo, Jesus Santamaria

**Affiliations:** a Institute of Nanoscience of Aragon (INA) and Department of Chemical Engineering and Environmental Technology , University of Zaragoza , C/Mariano Esquillor , s/n , I+D+i Building , 50018 , Zaragoza , Spain . Email: victorse@unizar.es ; Email: jesus.santamaria@unizar.es ; Fax: +34 976 761879 ; Tel: +34 876555441; b CIBER de Bioingeniería , Biomateriales y Nanomedicina (CIBER-BBN) , Centro de Investigación Biomédica en Red , C/Monforte de Lemos 3-5 , Pabellón 11 , 28029 Madrid, Spain; c Laboratorio de Microscopias Avanzadas (LMA) , Instituto de Nanociencia de Aragon (INA) , Universidad de Zaragoza , Mariano Esquillor I+D , 50018, Zaragoza , Spain

## Abstract

In this work we investigate the formation of PtAu monodisperse alloyed nanoparticles by ageing pure metallic Au and Pt small nanoparticles (sNPs), nanoparticle size <5 nm, under certain conditions. We demonstrate that those bimetallic entities can be obtained by controlling the size of the initial metallic sNPs separately prepared and by selecting their appropriate capping agents. The formation of this spontaneous phenomenon was studied using HR-STEM, EDS, ionic conductivity, UV-Vis spectroscopy and cyclic voltammetry. Depending on the type of capping agent used and the size of the initial Au sNPs, three different materials were obtained: (i) AuPt bimetallic sNPs showing a surface rich in Au atoms, (ii) segregated Au and Pt sNPs and (iii) a mixture of bimetallic nanoparticles as well as Pt sNPs and Au NPs. Surface segregation energies and the nature of the reaction environment are the driving forces to direct the distribution of atoms in the bimetallic sNPs. PtAu alloyed nanoparticles were obtained after 150 h of reaction at room temperature if a weak capping agent was used for the stabilization of the nanoparticles. It was also found that Au atoms diffuse towards Pt sNPs, producing a surface enriched in Au atoms. This study shows that even pure nanoparticles are prone to be modified by the surrounding nanoparticles to give rise to new nanomaterials if atomic diffusion is feasible.

## Abstract 2

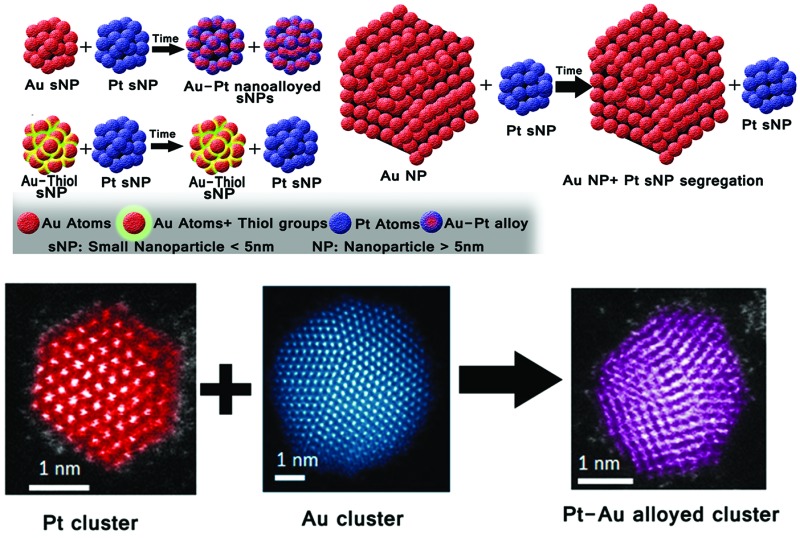
In this work we investigate the formation of PtAu monodisperse alloyed nanoparticles by ageing pure metallic Au and Pt small nanoparticles (sNPs), nanoparticle size <5 nm, under certain conditions.

## Introduction

Noble metal nanoparticles are interesting because in the nanoscale regime, somewhere between the bulk solid and the molecular states, they exhibit chemical and physical properties that are unique and significantly different from those of their corresponding bulk counterparts. In nanomaterials, surface atoms have a strong influence on the physico-chemical behavior of the material. Nevertheless, the enormous surface area-to-volume ratio of nanoparticles creates a greater excess of free energy in comparison with the lattice energy of the bulk, which may lead to surface instability.^1,2^ Capping agents, which adsorb on the particle surface, promote nanoparticle stabilization according to three pathways: electrostatic, steric and electrosteric.^3^ The nanoparticle size and shape dependent properties can be tailored by varying the type or amount of the stabilizer.^4^ The presence of a large number of surface atoms in clusters and the short diffusion paths involved can enhance mass transport inside a particle in a phenomenon known as “Spontaneous Alloying”. According to such a phenomenon a binary core–shell metal cluster rapidly becomes an alloyed cluster at room temperature, the key transformation factors being the size of the cluster, the nature of the bimetallic system studied and the heat of dispersion after mixing.^5–9^

An extensive study of spontaneous alloying has been carried out by Mori and co-workers, reporting the rapid alloying of Cu,^10^ Zn,^6^ Pb^11^ and Sb^12^ with gold. Other nanoparticulated bimetallic pairs are spontaneously alloyed at room temperature, including PdAg,^13^ PtNi^14^ and so on. Kinetic considerations indicate that, for the two metals alloying, the diffusion coefficient needs to be many orders of magnitude larger than that for the bulk material.^6,10^ In addition, it is still not well understood whether the alloying process is directed by surface diffusion alone or whether it requires additional startups such as defect creation at the interface or crystal facet changes. Molecular dynamics simulation has been used as a tool to gain knowledge of the diffusion mechanisms of spontaneous alloying, although the atomistic mechanism has not been fully elucidated.^15,16^ Furthermore, research on long-time dynamics beyond microseconds remains still unexplored in sNPs alloying.

AuPt nanoparticles have been reported to exhibit interesting properties, including a reduction of susceptibility to CO poisoning and enhancement of catalytic performance.^17–19^ Thus, understanding nanoscale alloying and phase segregation mechanisms is of great interest in nanoscale chemistry and its applications. It is accepted that the alloying or phase-segregation depends on a number of parameters, including the preparation method and support properties. For instance, AuPt nanoparticles prepared by microemulsion^20^ showed single-phase alloying but those prepared by the polyol method gave rise to segregated particles.^21^

**Scheme 1:**
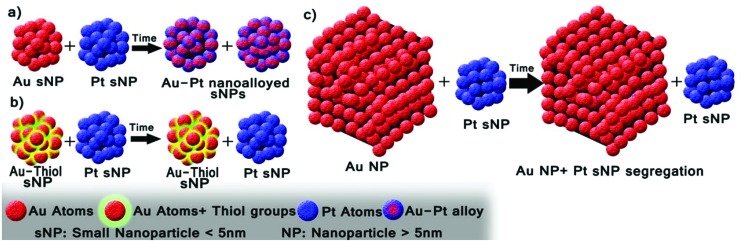
Schematic representation of the time alloying process. (a) Metal sNPs stabilized with weak ligands produce AuPt nanoalloys. (b) When Au sNPs are stabilized with thiol groups (a strong bond capping agent) Pt and Au sNPs remain segregated even if Pt sNPs have a weak capping agent. (c) When 20 nm size Au nanoparticles stabilized with weak capping agents are used the particles remain segregated even if Pt sNPs have a weak capping agent.

In this work, we focus on the synthesis of monodisperse AuPt small nanoparticles (sNPs), nanoparticle size <5 nm, obtained as aqueous suspensions at ambient temperature following a spontaneous alloying process. Nanoalloying was obtained depending on the nanoparticle sizes and the nature of the ligands stabilizing their surfaces. As a novelty, the alloying process was achieved starting from pure metal nanoparticles (Pt and Au) stabilized with weakly bound capping agents, rather than with their dissolved chemical precursors. In this case, the interaction in a dispersion of both nanoparticles with a particle size below 5 nm gives rise to AuPt bimetallic nanoparticles. However, the presence of strongly bonded capping agents, such as thiol groups, or the use of larger metal nanoparticles promotes the presence of unmixed entities instead of alloying (see Scheme 1).

## Experimental section

The experiments have been performed by the platform of Production of Biomaterials and Nanoparticles of the NANBIOSIS ICTS, more specifically by the Nanoparticle Synthesis Unit of the CIBER in BioEngineering, Biomaterials & Nanomedicine (CIBER-BBN).

### Chemicals

Tetrakis(hydroxymethyl) phosphonium chloride solution (THPC, 80 wt%, Aldrich), poly(ethylene glycol) methyl ether thiol (Mn 800, Aldrich), sodium citrate tribasic dehydrate (≥99%, Aldrich), chloroplatinic acid 8 wt% solution (H_2_PtCl_6_, Aldrich), tetrachloroauric acid trihydrate (HAuCl_4_·3H_2_O, Aldrich), and sodium hydroxide (NaOH, Aldrich) were all used as received.

### Synthesis of THPC gold small nanoparticles

The synthesis of THPC gold nanoparticles was performed by adding 3 mg of HAuCl_4_ to 15 mL of distilled water in a glass vial under magnetic stirring. Then 165 μL of a 1 M NaOH solution was added and, finally, 333 μL of a 65 mM THPC solution was added. The reaction mixture was stirred for 1 day at room temperature, protecting the mixture from light to prevent the photothermal decomposition of precursors.^22^ The resulting colloid was then stored in a refrigerator.

### Synthesis of THPC gold small nanoparticles stabilized with thiol-PEG

Au-THPC nanoparticles stabilized with thiol-PEG ligands were produced by adding 3 mg of HAuCl_4_ to 15 mL of distilled water in a glass vial under magnetic stirring. Then 165 μL of a 1 M NaOH solution and 24 mg of thiol-PEG were added. Finally, 333 μL of a 65 mM THPC solution was added. The reaction mixture was stirred for 4 days at room temperature, protecting the mixture from light to prevent photothermal decomposition. The resulting colloid was then stored in a refrigerator.

### Synthesis of citrate gold nanoparticles

The synthesis of citrate gold nanoparticles was performed by adding 4 mg of HAuCl_4_ to 10 mL of distilled water in a glass vial. In another vial, 60 mg of citrate were mixed with 15 mL of distilled water. 5 mL of gold solution was heated to 65 °C and then 5 mL of citrate solution were added. When a colour change was observed, 2.5 mL of the citrate solution were added. After 10 minutes, 5 mL of each solution were sequentially added. Finally, after 10 minutes under mixing, the remaining 2.5 mL of citrate solution were added. The obtained colloid was always stored in a refrigerator.

### Synthesis of platinum small nanoparticles

In a typical synthesis of platinum nanoparticles, 100 μL of H_2_PtCl_6_ 8 wt% solution was added to 15 mL of distilled water in a glass vial under magnetic stirring. Then 165 μL of a 1 M NaOH solution was added and, finally, 333 μL of a 65 mM THPC solution was added. The reaction mixture was stirred for 4 days at room temperature, protecting the mixture from light to prevent photothermal decomposition. The produced Pt nanoparticles were then stored in a refrigerator.

### Synthesis of PtAu small nanoparticles by the simultaneous addition of metal precursors

The synthesis of PtAu nanoalloyed particles was performed by adding 50 μL of H_2_PtCl_6_ 8 wt% solution and 1.5 mg of HAuCl_4_ to 15 mL of distilled water in a glass vial under magnetic stirring. Afterwards, 165 μL of a 1 M NaOH solution were added and, finally, 333 μL of a 65 mM THPC solution was added. The mixture was kept under stirring for 4 days, protecting the resulting colloid from light to prevent photothermal-directed reactions at ambient temperature. Their characterization was performed by UV-Vis spectrometry.

### Synthesis of PtAu alloyed small nanoparticles stabilized with THPC by a physical mixture

Bimetallic PtAu nanoparticle synthesis was carried out by mixing 5 mL of as-made Pt nanoparticles with 5 mL of as-made THPC Au nanoparticles. The mixture was kept under stirring, protecting from light to prevent photothermal-directed reactions, at ambient temperature. Their characterization was performed by UV-VIS spectrometry.

### Synthesis of citrate PtAu nanoparticles by physical mixture

The synthesis of bimetallic particles was carried out by mixing 5 mL of as-made Pt nanoparticles with 5.9 mL of as-made citrate Au nanoparticles. The mixture was kept under stirring, protected from light to prevent photothermal decomposition, at ambient temperature. Their characterization was performed by UV-Vis spectrometry.

### Conductometry characterization

Conductometry characterization was performed with a portable conductivity meter Crison 524, with the E.C. Cell 5290 at room temperature.

### Electrochemical characterization

The electrochemical experiments were carried out in a standard three-electrode system controlled using NOVA 1.8 (Autolab PGSTAT 101) with glassy carbon and reversible hydrogen electrodes (RHE) as counter and reference electrodes, respectively.

The working electrode was a thin layer of Nafion-impregnated-metal cast on a vitreous carbon disk. This electrode was prepared by ultrasonically dispersing a metal suspension in MilliQ water and Nafion (Aldrich, 15% in water). The suspension of nanoparticles was deposited onto the carbon disks and dried at room temperature.

The room temperature cyclic voltammetry tests were recorded between 0 and 1.1 V for the Pt NP sample and between 0 and 1.6 V for samples that contained Au at 50 mV s^–1^, in nitrogen-purged H_2_SO_4_ (0.5 M) as the electrolyte. Several cycles at a high scan rate (500 mV s^–1^) were performed before electrochemical measurements in order to clean the metal surface.

### Electron microscopy characterization

Preliminary electron microscopy observations were carried out using a T20-FEI microscope with a LaB_6_ electron source fitted with a “SuperTwin®” objective lens allowing a point-to-point resolution of 2.4 Å. Aberration corrected scanning transmission electron microscopy (C_s_-corrected STEM) images were acquired using a high angle annular dark field detector in a FEI XFEG TITAN electron microscope operated at 300 kV equipped with a CETCOR C_s_-probe corrector from CEOS Company allowing formation of an electron probe of 0.08 nm. The geometric aberrations of the probe-forming system were controlled to allow a beam convergence of 24.7 mrad half-angle to be selected. Elemental analysis was carried out with an EDS (EDAX) detector which allows performing EDS experiments in the scanning mode.

## Results and discussion

### Spontaneous nanoalloying of THPC-stabilized Au and Pt sNPs

**Fig. 1:**
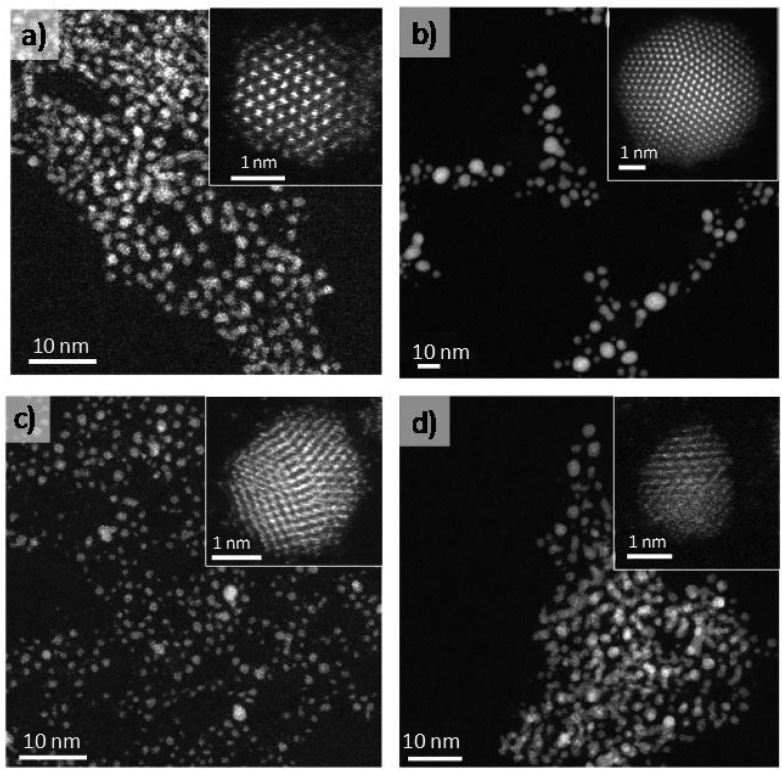
*C*_s_-corrected STEM-HAADF images. Insets are high-magnification STEM-HAADF images. (a) Pt sNPs, (b) Au sNPs, (c) PtAu nanoalloyed sNPs produced by simultaneous addition of metal precursors and (d) PtAu nanoalloyed sNPs obtained by pure metal sNPs mixing after 10 days at room temperature.

**Fig. 2:**
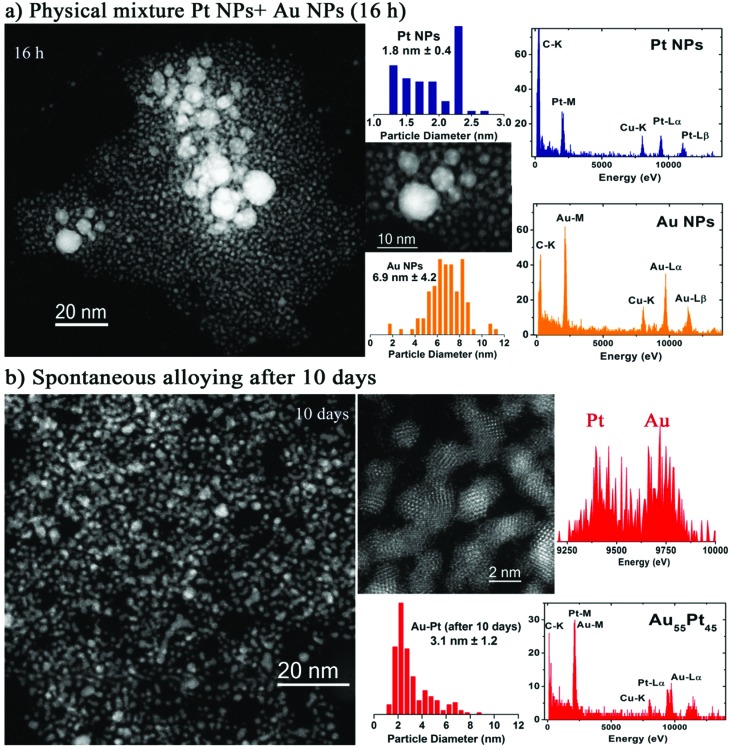
*C*_s_-corrected STEM-HAADF images, particle size distribution and representative EDS analysis of single PtAu sNPs obtained by the physical mixture of Au and Pt sNPs stabilized with THPC at several times: (a) 16 h and (b) spontaneous alloying after 10 days.

In a previous study,^23^ we showed that the use of tetrakis-(hydroxymethyl) phosphonium chloride solution (THPC) as the simultaneous reducing and stabilizing agent at room temperature in aqueous phase renders a variety of monometallic, bimetallic and trimetallic noble metal clusters. Tris(hydroxymethyl) phosphine oxide (THPO) is the cleavage product of THPC in alkaline solution, and it adsorbs onto the surface of the colloidal NPs as a protecting ligand during the reduction of noble metal precursors.^24^ Fig. 1a and b show a representative selection of the spherical aberration (*C*_s_) corrected STEM-HAADF images of Pt and Au small nanoparticles (sNPs) obtained at room temperature after 4 days of reaction time following this approach. In the case of the Pt clusters, well-crystallized structures of 1.8 nm were observed which could be indexed matching the *Fm*3*m* space group of Pt. On the other hand, gold nanoparticles with a relatively wide size distribution between 4 and 6 nm (Fig. 1b), with decahedral morphology, presented a certain tendency to coarsen at room temperature though this process could be avoided if they were kept under 4 °C.^23,25^ PtAu bimetallic sNPs were obtained if platinum and gold precursors were simultaneously mixed before the reduction process was induced by the THPC decomposition under alkaline conditions (see Fig. 1c). The particle size, chemical composition and atomic distribution were well controlled using this easy synthesis procedure.^23^ Finally, when preformed Pt and Au sNPs were mixed in equimolar concentrations, Au sNPs tended to coarse while Pt sNPs were stable (Fig. 2a). After several days of aging at room temperature, PtAu nanoalloyed sNPs with a Pt/Au atomic ratio of 1.2 were obtained (Fig. 2b).

Pt sNPs were stable at room temperature for several months (see Fig. S1 in ESI†), but as already mentioned gold sNPs suffered from coarsening and large nanoparticles with a broad size distribution were obtained after keeping the colloid for several days at room temperature. These interesting facts show that THPC enables the production of stable Pt and PtAu sNPs but its stabilizing role is inefficient in keeping the size of Au sNPs stable for a long time at room temperature. These results are consistent with the observations of Luo *et al.*,^24^ who noted that a weaker interaction of THPO on the surface of Au nanoparticles correlated with a higher catalytic activity compared to strongly bond capping agents. The particle size distribution of Pt, Au and PtAu sNPs was continuously modified along the ageing time (Fig. 2). After 10 days of ageing, the particle size distribution of the remaining sNPs is similar (Fig. 1d and 2b), implying that the interaction of Pt and Au atoms is good enough to avoid coarsening.

*C*_s_-corrected STEM measurements demonstrated that the AuPt nanoparticles (3.1 nm) obtained from as-made pure counterparts have a slightly larger mean particle size than the Au (4–6 nm)|Pt (1.8 nm) starting sNPs (see STEM images in Fig. 1d and 2b), but no single Au nanoparticles were observed (see EDS spectra in Fig. 2b). The particle size distribution was very similar to the Pt as-made sNPs, inferring that the interaction between sNPs was fast enough to prevent gold sNPs from a high grade of coarsening and the consequent formation of large Au nanoparticles.

The microstructure of the bimetallic nanoparticles was also investigated using *C*_s_-corrected STEM. The chemical composition was studied by EDS analysis proving the presence of both elements. Unfortunately, due to the similar atomic number between both elements, no contrast difference was observed by the HAADF *z*-contrast in the nanoparticles which did not make possible to establish an elemental distribution (Fig. 2b). The STEM analyses of individual metal sNPs obtained after 10 days of ageing contain both elements, being difficult to elucidate the distribution of Au and Pt atoms.

The above results show that mixing of separately prepared Pt and Au sNPs in solution phase gives rise to sNPs AuPt nanoparticles whose properties are similar to those obtained from the simultaneous mixing of precursors. This represents a new approach to modify the chemical composition of nanomaterials. Furthermore, this implies that nanoparticle ageing is an important variable to consider in catalytic reactions, since the Pt/Au catalyst composition can be modified from that existing at the beginning of the process if the catalyst were composed of separate Au and Pt nanoparticles.

### Particle size and ligand influence on spontaneous nano-alloying

**Fig. 3:**
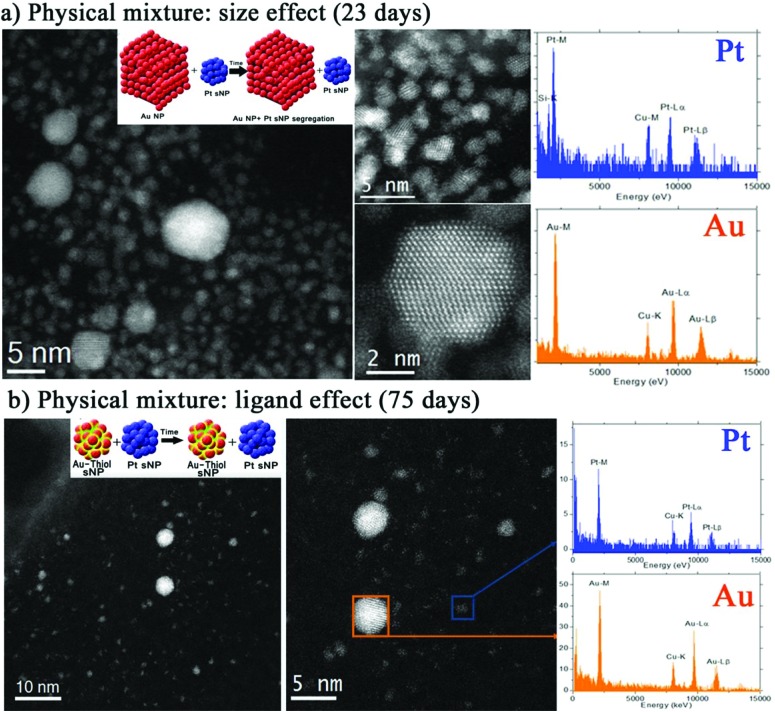
*C*_s_-corrected STEM-HAADF micrographs and representative EDS analysis of PtAu colloid obtained after the physical mixture of Pt sNPs stabilized with THPC ligands and Au nanoparticles stabilized with: (a) citrate ligands, ageing time = 23 days and (b) thiol-PEG ligands, ageing time = 75 days.

To further investigate the inter-diffusion time of as-made Pt and Au sNPs after mixing and correlate this phenomenon with the particle size and surface energy of sNPs, gold nanoparticles stabilised with citrate ions, a weak capping agent, and with a mean particle size of 20 nm were equally mixed with 1.5 nm Pt sNPs. After more than 20 days of ageing, the STEM-EDS spectrum clearly shows the presence of pure Pt sNPs and Au nanoparticles without alloying (Scheme 1c and Fig. 3a). These findings imply that the particle size of the starting nanoparticles is crucial in modifying the chemical composition of the resulting nanomaterials.

The same findings were observed if as-made Pt and Au sNPs were modified with a stronger capping agent than THPC. In this case, THPC stabilized Au sNPs were functionalized with thiol-PEG ligands and mixed in solution phase with Pt sNPs. The STEM-EDS spectrum of clusters aged after 75 days shows the presence of pure metallic entities only, without any intermixing between clusters or any change in particle size distribution (Fig. 3b and Scheme 1-b). The same observation (unchanged particle size distribution) was made in pure Au sNPs functionalized with thiol-PEG ligands after the same ageing time, which implies that thiol groups are strongly attached to the Au sNPs surface, preventing any atoms from surface diffusion and then blocking the coarsening.

The binary phase diagrams of bulk Au–Pt materials exhibit a large miscibility gap (20–90%) with limited solubility between the two face centered cubic (fcc) materials at ambient temperature.^26^ This would indicate that Au and Pt alloying is not feasible, but it is not supported by the experimental evidence that, in contrast, indicates that Au and Pt alloying occurs at the nanoscale as compared with bulk AuPt materials.^19^ On the other hand, there is a small crystal lattice mismatch between both metals (<5%),^27^ which favors the formation of Au–Pt bimetallic nanoparticles. According to theoretical calculations, the heat of formation of AuPt nanoparticles is negative if the nanoparticle size is below 6 nm.^28^ This implies that the formation of AuPt nanoparticles is not thermodynamically favorable to nanoparticles larger than 6 nm or bulk AuPt materials. Consequently, it seems reasonable that Au–Pt inter-diffusion was favored when Pt sNPs were mixed with 2–4 nm Au clusters rather than with 20 nm ones.

### Spectroscopic and electrochemical characterization of spontaneous nanoalloyed particles

The electronic microscopy characterization has confirmed a clear process of spontaneous alloying of Au and Pt sNPs. However, the number of entities analysed are limited to local areas. Then, bulk characterization is also required to confirm the phenomenon reported herein. Considering the difficulties and limitations of preparing a considerable amount of nanoparticles, UV-VIS spectroscopy and electrochemical analyses were selected as the most appropriate techniques to elucidate the spontaneous nanoalloying at a bulk scale.

**Fig. 4:**
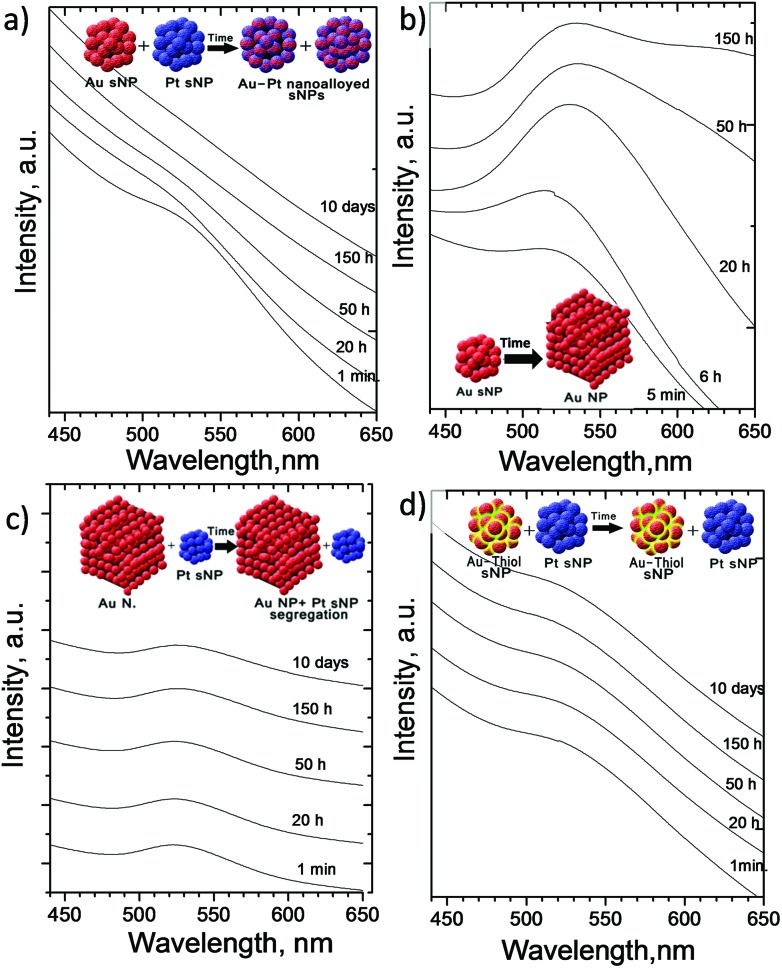
UV-Vis absorption spectra along time of: (a) the physical mixture of as-made Au and Pt sNPs stabilized with THPC. (b) Au stabilized with THPC sNPs. (c) The physical mixture of as-made 20 nm Au nanoparticles stabilized with citrate and Pt sNPs stabilized with THPC; (d) the physical mixture of as-made Au sNPs stabilized with thiol-PEG ligands and Pt sNPs stabilized with THPC.

Fig. 4a depicts the UV-VIS characterization of the colloid resulting from the physical mixture of as-made Pt and Au sNPs stabilized with THPC. It confirms that the typical surface plasmon absorption peak of Au nanoparticles at the wavelength of 520 nm decreases with time and disappears after 150 hours of contact in the liquid phase (see Fig. 4a). This phenomenon is consistent with a previous observation where the presence of a group 10 metal (d^8^s^2^) in the bimetallic nanoparticles suppressed the surface plasmon resonance energies of group 11 metals (d^10^s^1^)^29^ and implied the formation of Au/Pt bimetallic nanoparticles. This finding is in full agreement with the local STEM-EDS results where Au and Pt atoms were located in the same sNP. The surface free energy for Pt is larger than that of Au.^28^ This difference promotes the diffusion of Au over Pt nanoparticles, which is consistent with the fact that the final size of aged AuPt bimetallic nanoparticles was closer to the Pt sNPs rather than the Au ones. Nevertheless the suppression of the surface plasmon absorption implies that Au is not totally segregated at the shell of AuPt clusters. Otherwise, an Au shell should have been formed and a new plasmon absorption peak observed.^30^ Then it seems reasonable to say that there is a re-distribution of Au and Pt in the nanoparticle, which evolves in such a way that the Au concentrates in the region near the outer shell whereas the Pt concentrates in the core region. The gradual redistribution of Au should explain the slow suppression of the Au surface plasmon peak with time during the formation of AuPt clusters from as-made Pt and Au sNPs.

The low stability at room temperature of Au sNPs stabilized with THPC is reflected in Fig. 4b. The surface plasmon absorption peak increases in intensity and red-shifts with time, which implies that Au sNPs increase in size and aggregate.^25^ However, when Pt sNPs are present, gold atoms coming from the Au sNPs tend to diffuse to Pt sNPs to form bimetallic AuPt clusters rather than aggregate into larger nanoparticles. On the other hand, if 20 nm Au nanoparticles are physically mixed with Pt sNPs, the formation of the AuPt bimetallic sNPs is not thermodynamically favored^28^ (heat of formation is positive) and nanoparticle segregation is maintained. In this case, Au atoms are stable at the surface of the Au nanoparticles and their size is vaguely modified with time. This statement is supported by the lack of evolution of the surface plasmon resonance peak of the physical mixture of 20 nm Au nanoparticles with Pt sNPs (Fig. 4c).

Regarding the findings observed with the STEM-EDS local analysis of the physical mixture of Pt sNPs stabilized with THPC and Au sNPs stabilized with thiol-PEG, Fig. 4d also confirms that the electronic properties and then the morphology and chemical composition of Au sNPs are not modified along time. The characteristic surface plasmon resonance peak of Au sNPs after 10 days is similar to that shown by the as-made sNPs.

**Fig. 5:**
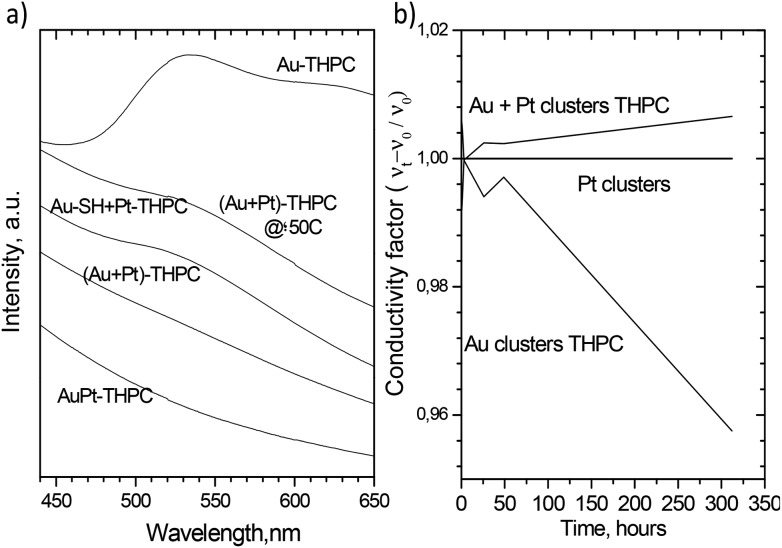
(a) Comparison of UV-Vis absorption spectra of different physical mixtures after 150 h of mixing: (i) as-made Au sNPs stabilized with THPC, (ii) as-made Au and Pt sNPs stabilized with THPC and aged at 50 °C, (iii) as-made Au and Pt sNPs stabilized with thiol groups and Pt stabilized with THPC, (iv) as-made Au and Pt sNPs stabilized with THPC and aged at room temperature and (v) PtAu sNPs obtained after mixing of precursors. (b) Conductivity of colloid dispersions with time in Au and Pt sNPs stabilized with THPC and the physical mixture of as-made Au and Pt sNPs stabilized with THPC. The conductivity factor was calculated referred to the solvent, Milli-Q water.

Fig. 5a shows the UV-VIS spectra of Au–Pt nanoparticles formed under different conditions after 150 h of mixing time. Apart from the previous findings, it can be highlighted that the sNPs produced after the simultaneous reduction of the Au and Pt precursors have no surface plasmon resonance. In this case, Au atoms are stable at the surface of the Au nanoparticles and their size is vaguely modified with time. This statement is supported by the lack in evolution of the plasmon peak. This electro-optical behaviour is the same as that achieved with the physical mixture of Au and Pt sNPs stabilized with THPC. Nevertheless, if this physical mixture of Au and Pt stabilized with THPC is kept at 50 °C rather than at room temperature, the surface resonance plasmon peak of Au sNPs decreases but does not disappear and a mixture of pure gold nanoparticles and bimetallic sNPs was found (see Fig. S2 in the ESI†). It would be expected that the temperature increase could accelerate the atom inter-diffusion between Au and Pt clusters, but it seems that gold coarsening is also accelerated and there is a competitive mechanism to produce bimetallic Au–Pt and Au nanoparticles.

The conductivity of the Au and Pt sNPs stabilized with THPC and the physical mixture of both sNPs was tracked along time to measure the quantity of ionic species in solution during the metal cluster transformation. Fig. 5b shows that the conductivity of Pt sNPs dispersion does not change with time, which confirms the stability already mentioned of Pt sNPs stabilized with THPC. On the other hand, the conductivity trend of Au sNPs in dispersion is in agreement with the UV-VIS and microscopy characterization, since the presence of ionic species diminished with time. The total ionic conductivity will be attributed to the capping agents (*i.e.* bivalent citrate anions and phosphine oxide), Cl^–^ generated during the reduction of AuCl_4_^–^ and OH^–^, as well as counterions (H^+^, Na^+^). Au ions [Au^+^Cl^2–^] on the surface of the Au^31^ nanoparticles which are probably incorporated into the growing AuPt alloy, decreasing the total ionic conductivity. This behaviour could be supported by the idea of an increase in the particle size of Au nanoparticles and then the decrease of Au charged species. Finally, the trend of Au and Pt sNPs physically mixed is completely the opposite of that followed by Au sNPs and the concentration of ionic species increased with time. This finding could be explained by the continuous diffusion of Au species from Au sNPs to Pt sNPs and the evolution over time to form the Au–Pt bimetallic sNPs, as well as the change in the electronic properties of the formed sNPs.

**Fig. 6:**
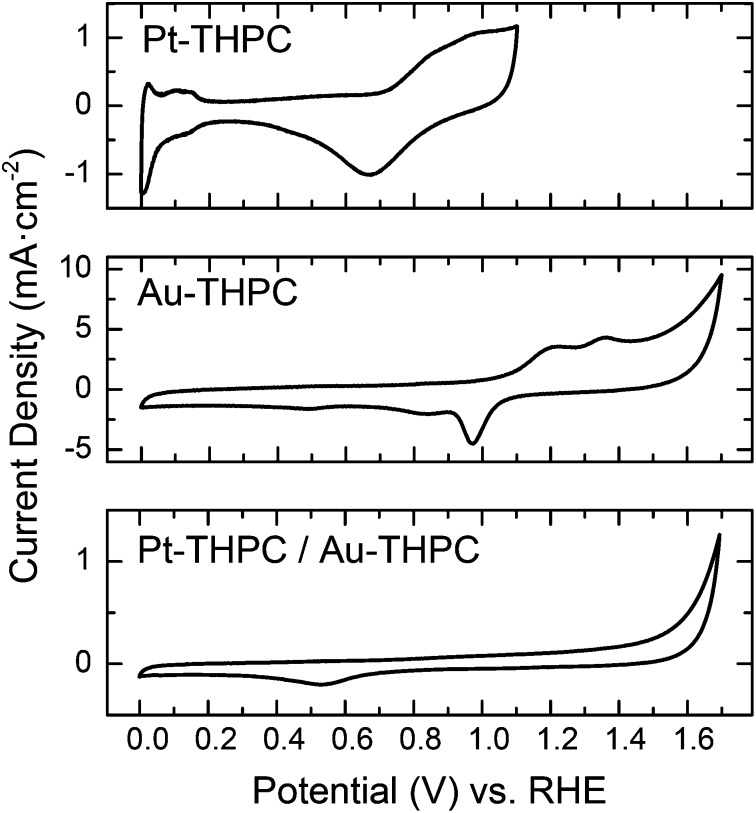
Cyclic voltammetry (sweep rate 50 mV s^–1^) at 298 K and 0.5 M H_2_SO_4_ of Pt and Au sNPs stabilized with THPC and physically mixed and analysed after 15 days. Cyclic voltammograms show that the onset of oxide adsorption on Au is positively shifted by more than 600 mV *vs.* Pt, indicating that Pt is much more oxophilic than Au.

The surface properties associated with the nanoscale alloying or phase segregation of the bimetallic nanoparticles can also be addressed by measurements of the electrochemical properties. In fact, the electrochemical properties of Au and Pt nanoparticles are different since Au nanoparticles are inactive for hydrogen adsorption.^32^ Then, the electrochemical characterization of the bimetallic Au–Pt sNPs obtained after the physical mixture of pure sNPs can give some insight into the atomic distribution of Au and Pt. The cyclic voltammograms (CVs) of the monometallic and bimetallic sNPs in 0.5 M H_2_SO_4_ are displayed in Fig. 6. In the CVs of the Pt sNPs, characteristic current peaks that can be assigned to the hydrogen adsorption/desorption on the Pt surface are identified at around 0 and 0.2 V *vs*. RHE, indicating the effective removal of surfactants and organic materials from the surface of the Pt sNPs by the previous electrochemical cleaning process. Oxidation/reduction of Pt events can be located at potentials around 0.6–1.1 V *vs*. RHE in the same voltammogram.

The voltammogram associated with the Au THPC-stabilized sNPs only displays current peaks at potentials higher than 0.9 V related to the oxidation/reduction Au surface. Hamelin *et al.* ^33^ identified the shape of the voltammogram plot with a preferential orientation in Au clusters. This kind of CV graph can be associated with Au(111). On the one hand, Au THPC-stabilized sNPs showed no peak associated with hydrogen adsorption/desorption. Thus, any hydrogen adsorption and desorption feature on the CV curves of the AuPt samples should be associated with the adsorption and desorption of hydrogen atoms on the Pt. On the other hand, any event at high potential should be associated with the oxidation/reduction in the Au surface. AuPt bimetallic sNPs obtained after the physical mixture of pure sNPs stabilized with THPC show no characteristic peaks (see Fig. 6). This fact can be explained if we consider that the individual metallic nanoparticles have lost their own identity, with Pt being partially encapsulated by Au atoms, in agreement with the above TEM studies and UV-VIS spectra. Besides, this finding implies that the AuPt bimetallic clusters are enriched with Au atoms at the surface and this is consistent with the fundamental properties of Au and Pt, since it is always expected to have diffusion of Au atoms towards and over Pt surface atoms due to the difference in surface segregation energies.^34,35^ This altered surface segregation enriched with Au atoms was not expected due to the given electrochemical conditions during the physical mixture under air atmosphere (oxidant conditions). According to the redox potential, Pt is easier to be oxidized than Au (Fig. 6), which should provide the driving force for Pt atoms to stay on the surface in a highly oxophilic environment. This fact implies that the galvanic replacement reaction of Au by Pt is neglected. This result is in agreement with the reduction potentials of Au (1.56 V *vs.* SHE) and Pt (1.18 V *vs.* SHE). For this reason, it seems that there is a counterbalance between two opposing forces, namely the rather strong interaction between Pt and surface oxides on the one hand and the tendency of Au to segregate over Pt on the other hand. However, in this case, the thermodynamic driving force for Au to segregate to the surface of a Pt lattice is directing the formation of bimetallic AuPt sNPs.

**Fig. 7:**
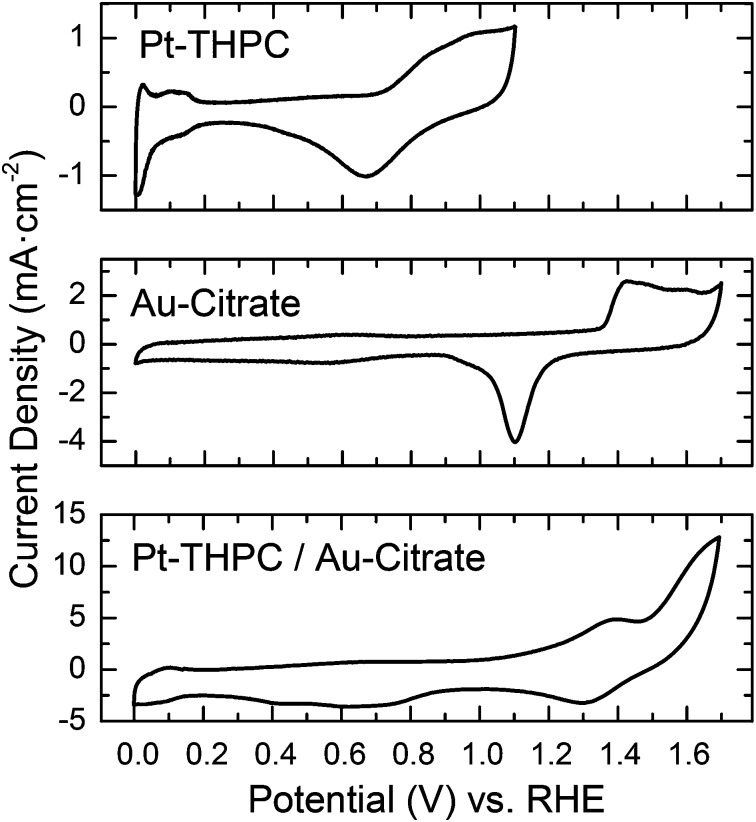
Cyclic voltammetry (sweep rate 50 mV s^–1^) at 298 K and 0.5 M H_2_SO_4_ of Pt sNPs stabilized with THPC, 20 nm Au nanoparticles stabilized with citrate ions, and sNPs physically mixed and analysed after 15 days. Cyclic voltammograms show that the onset of oxide adsorption on Au is positively shifted by more than 600 mV *vs.* Pt, indicating that Pt is much more oxophilic than Au.

In Fig. 7 the 20 nm citrate-Au nanoparticle voltammogram is depicted. Again, the characteristic peaks associated with redox events in the Au surface appear at high potentials between 0.9 and 1.7 V, although the shape of the plot has changed. This kind of voltammogram can be attributed to Au(110) facets.^33^ Regarding the electrochemical characterization of the nanoparticles resulting from the physical mixture of 20 nm citrate-Au nanoparticles and Pt stabilized sNPs, CVs depicted in Fig. 7 show the presence of hydrogen adsorption/desorption in the range of 0–0.2 V *vs*. RHE. This fact was expected, since EDS characterization clearly showed the presence of segregated Pt and Au sNPs (see Fig. S2 in the ESI†). Then, the peaks associated with hydrogen adsorption/desorption can be attributed to the presence of pure Pt sNPs. In addition, the presence of the current peaks at high potential (*E* > 1.4 V) is attributed to both, OH adsorption on Au sites and the O_2_ evolution on Pt sites. Thus the CV in Fig. 7 is fully consistent with a scenario where Au and Pt sNPs have been mixed but have not lost their own identity (*i.e.*, they have not been alloyed).

## Conclusions

In summary, we have shown, using local and bulk characterization techniques, that by controlling the size of metallic sNPs separately prepared and by selecting their capping agents, bimetallic entities can be obtained. Depending on the type of capping agents and the size of the Au sNPs used, three different materials were obtained: (i) AuPt bimetallic sNPs, where the surface is enriched with Au atoms, (ii) non-alloyed Au and Pt sNPs and (iii) a mixture of bimetallic nanoparticles and segregated Pt sNPs and Au nanoparticles. The role of the capping agents used is essential to determine the fate of metal sNPs with a size below 5 nm. Thus, a strongly bonded capping agent should be selected to preserve the segregation after the synthesis of metal sNPs. Alternatively, weakly adsorbing species should be used in conjunction with a small particle size in order to promote the formation of nanoalloys. Surface segregation energies and the nature of the reaction environment are the driving forces directing the distribution of atoms in the bimetallic sNP. The strategy reported here could also be generalized to connect fundamental studies and novel nanomaterial synthesis for advanced catalysis and other applications. Finally, these results also highlight the fact that under mild conditions pure sNPs can be transformed into nanoalloys, which can be of great help in order to properly interpret the obtained results in post-reaction catalyst characterization.
