# Supplementary material for: Spontaneous formation of Au–Pt alloyed nanoparticles using pure nano-counterparts as starters: a ligand and size dependent process
Source: Nanoscale. 2015 May 19;7(22):10152–61. doi: 10.1039/c5nr01819f (PMC4447060; doi:10.1039/c5nr01819f)
Supplement: Supplementary file 1 [file NR-007-C5NR01819F-s001.pdf]

Electronic Supplementary Material (ESI) for Nanoscale.
This journal is © The Royal Society of Chemistry 2015

Supporting Information

Spontaneous formation of Au-Pt alloyed nanoparticles using pure nano-counterparts as starters: a ligand and size dependent process

Laura Uson, Victor Sebastian, Alvaro Mayoral, Jose Luis Hueso, Adela Eguizabal, Manuel Arruebo and Jesus Santamaria.

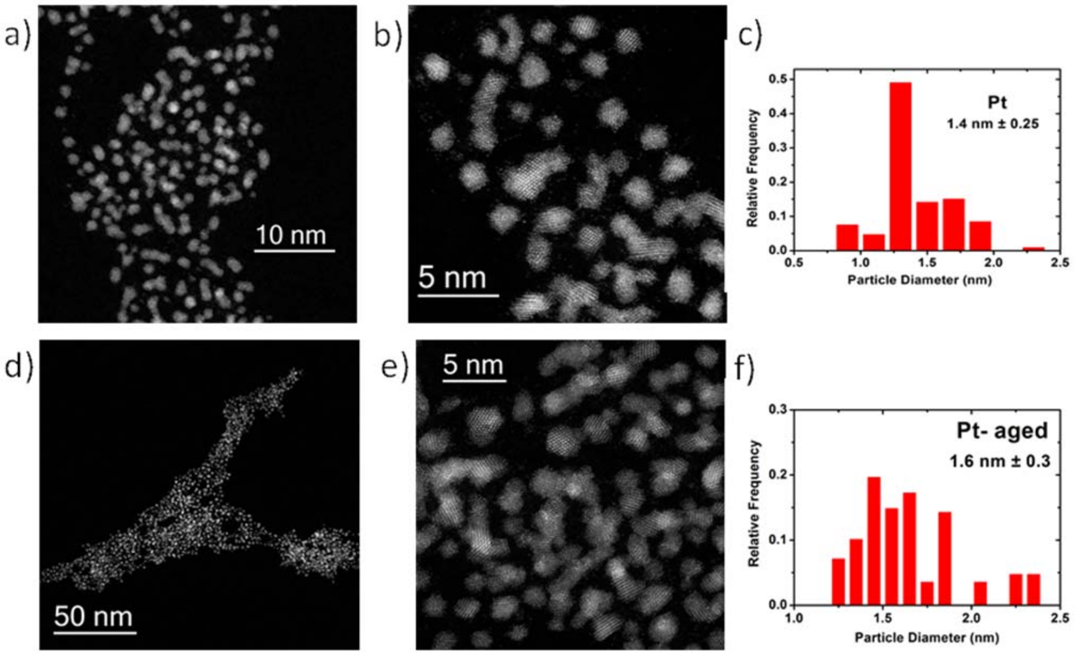

Figure S1. STEM-HAADF micrographs of Pt clusters. a) Pt clusters as prepared, b) Detail image of Pt clusters as prepared. It can be observed the nanocrystal lattice and a well-crystallization. c) Particle size distribution of Pt clusters as prepared. d) and e) Pt clusters after an ageing process of 4 months. The clusters keep a well-crystallized structured. f) Particle size distribution of Pt clusters aged during 4 months.

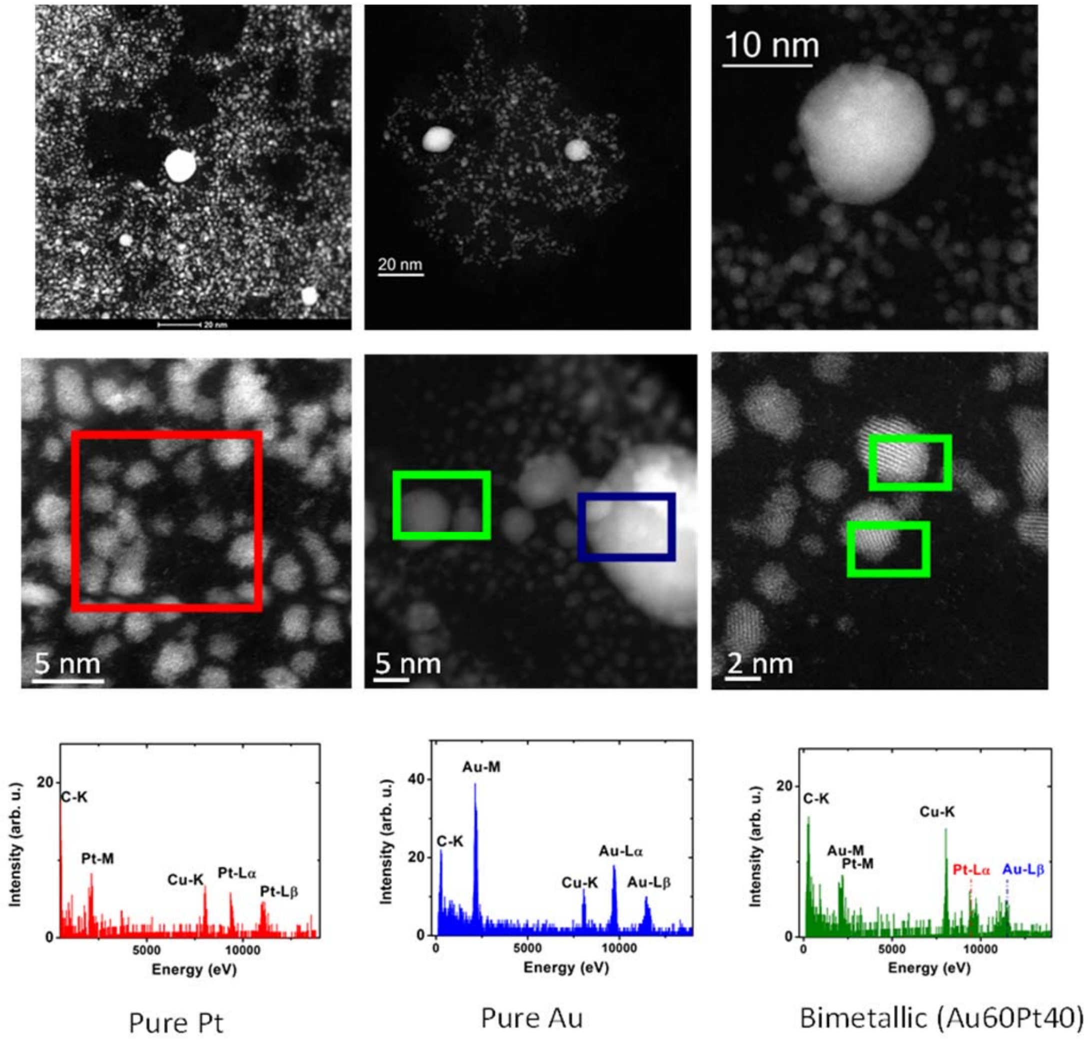

Figure S2. C_s-corrected STEM-HAADF micrographs and EDS analysis of PtAu colloid obtained by the physical mixture of Au and Pt clusters stabilized with THPC ligands after 150 h of mixing at 50ºC.
